# CaCO_3_ Polymorphs Used as Additives in Filament Production for 3D Printing

**DOI:** 10.3390/polym14010199

**Published:** 2022-01-04

**Authors:** Lucie Zárybnická, Radek Ševčík, Jaroslav Pokorný, Dita Machová, Eliška Stránská, Jiří Šál

**Affiliations:** 1 Institute of Theoretical and Applied Mechanics of the Czech Academy of Sciences, Prosecká 809/76, 190 00 Praha, Czech Republic; zarybnicka@itam.cas.cz (L.Z.); machova@itam.cas.cz (D.M.); 2 Department of Technical Studies, College of Polytechnics Jihlava, Tolstého 16, 586 01 Jihlava, Czech Republic; 3 Department of Civil Engineering, Faculty of Technology, Institute of Technology and Business, Okružní 517/10, 370 01 Česke Budejovice, Czech Republic; jaroslav.pokorny@mail.vstecb.cz (J.P.); sal@mail.vstecb.cz (J.Š.); 4 MemBrain s.r.o., Pod Vinicí 87, 471 27 Stráž pod Ralskem, Czech Republic; eliskastr@seznam.cz

**Keywords:** 3D printing, FFF, filament, polypropylene, additives, CaCO_3_ polymorphs, mechanical properties

## Abstract

Nowadays, additive manufacturing—also called 3D printing—represents a well-established technology in the field of the processing of various types of materials manufacturing products used in many industrial sectors. The most common type of 3D printing uses the fused filament fabrication (FFF) method, in which materials based on thermoplastics or elastomers are processed into filaments. Much effort was dedicated to improving the properties and processing of such printed filaments, and various types of inorganic and organic additives have been found to play a beneficial role. One of them, calcium carbonate (CaCO_3_), is standardly used as filler for the processing of polymeric materials. However, it is well-known from its different applications that CaCO_3_ crystals may represent particles of different morphologies and shapes that may have a crucial impact on the final properties of the resulting products. For this reason, three different synthetic polymorphs of CaCO_3_ (aragonite, calcite, and vaterite) and commercially available calcite powders were applied as fillers for the fabrication of polymeric filaments. Analysis of obtained data from different testing techniques has shown significant influence of filament properties depending on the type of applied CaCO_3_ polymorph. Aragonite particles showed a beneficial impact on the mechanical properties of produced filaments. The obtained results may help to fabricate products with enhanced properties using 3D printing FFF technology.

## 1. Introduction

The use of three-dimensional (3D) printing is nowadays frequently applied technology in various industry sectors, including civil engineering, biotechnology, and automotive [1,2,3,4,5,6,7,8,9]. Several additive technology techniques were developed and are widely used, such as stereolithography (SLA), fused filament fabrication (FFF), poly-jet, selective laser melting (SLM), selective laser sintering (SLS), direct metal laser sintering (DMLS), and laminated object manufacturing (LOM). The selection of a particular technique is essential to design products of required parameters at the desired cost of materials—the most frequently used additive technologies can be sorted according to their ascendant financial requirements as follows LOM < FFF < SLA < SLS < DMLS/SLM [10].

FFF is one of the most often used additive technology due to its low energy consumption and possibilities of printing products with complex shapes [11]. It belongs to so-called bottom-up methods that are producing one layer at a time, and the final 3D structured products are fabricated by gradual accumulations of these 2D layers [12]. Further description of FFF technology may be found e.g., in [13,14] and references therein. Polylactic acid (PLA) [15,16,17,18,19], acrylonitrile butadiene styrene (ABS) [20,21,22,23,24], polyethylene terephthalate (PET) [25], polyethylene terephthalate glycol (PET-G) [26,27,28], polypropylene (PP) [29,30], and viscoelastic thermoplastic elastomers like thermoplastic polyurethane (TPU) [31,32] belong to the most common thermoplastics applied in FFF technology.

The properties of filaments may be modified by the usage of different additives based on the character of the polymeric material. Among the most widely used additives affecting the life of the polymeric material are light stabilizers (like UV absorbers, photooxidation inhibitors), antioxidants, flame retardants, or thermal stabilizers. The second group is additives affecting polymeric properties, such are antistatic, lubricants, fillers, pigments, and blowing agents [33,34,35,36,37,38,39].

As another suitable additive, calcium carbonate (CaCO_3_) can be considered. CaCO_3_ may form three anhydrous crystalline polymorphs—thermodynamically most stable calcite, and metastable vaterite and aragonite. It may be present also in hydrated crystal forms (ikaite (CaCO_3_·6H_2_O), monohydrate (CaCO_3_·H_2_O), hemihydrate (CaCO_3_·½H_2_O), and as an amorphous phase (amorphous calcium carbonate (ACC)) [40]. CaCO_3_ polymorph exhibits particles with different morphology and properties like physical–mechanical performance [41]. In general, it was shown in [42] that different particle‘s morphologies affected tensile strength of powders. Furthermore, CaCO_3_ is frequently used as a filler [43] because of its unique properties such as low toxicity, biological inertness, good dispersion within the polymer matrix, and low moisture content [44,45].

In the case of polymers in which CaCO_3_ is used, for example, as a filler in polyvinyl chloride (PVC), these reach increased rigidity and flexibility. Due to its white color, it can be used as a pigment, which is comparable to TiO_2_, but it is cheaper [46]. CaCO_3_ also provides high brightness and gloss and can thus replace lead-based stabilizers with a calcium/zinc system. In polypropylene, the application of CaCO_3_ (usually around 10 wt %) increases stiffness and resistance to weathering [47]. It is also used as a filler in unsaturated polyesters for the preparation of non-shrinkable structures [48,49]. Calcium carbonate belongs to the class of isomeric filler—the usage of smaller particles results in better adhesion to the matrix. Particles with a higher specific surface area have been identified to have a beneficial effect on the modulus of elasticity [50].

This work aimed to investigate the effect of different crystalline anhydrous polymorphs of CaCO_3_—namely, aragonite, calcite, and vaterite—exhibiting different properties, such as morphology, on the fabrication of filaments composed of random polypropylene copolymer using the FFF technique. The aim is to get the insight of their effect on physical-mechanical properties of resulted filaments which may find application in 3D printing.

## 2. Experimental Part

### 2.1. Materials

Polypropylene random copolymer (PPR) product Lumicene MR60MC2 (Total PetroChemicals & Refining S.A./N.V., Bruxelles, Belgium) in the shape of pellets was used as received. Calcite and vaterite were synthesized pure (≥99 wt %) using the mixing of two concentrated salt solutions, as further described in [51]. Aragonite was synthesized with a minor amount of calcite (≤4.7 wt %) following the procedure described in [52]. The quantitative phase analysis of X-ray diffraction patterns using Rietveld refinement confirming the purity of synthetized CaCO_3_ polymorphs was reported in [41]. To compare the synthesized product with commercially available one, also calcite available on the market (min. 95%, Lach-Ner, Ltd., Neratovice, Czech Republic) was used for filament fabrication.

### 2.2. Filaments Preparation

The mixed granulates of PPR Lumicene MR60MC2 with 5 wt % of the CaCO_3_ polymorphs were carried out on an extruder (HAAKE PolyLab OS Rheo Drive 16, ThermoScientific, Waltham, MA, USA) with a PTW 24/28 twin screw (cumulatively maintained at 2.5 kg·h^−1^, 60 rpm extruder, temperature profile 180–160 °C). Then, granulates were processed in a hydraulic press (ZHOT, Presshydraulika, Opava, Czech Republic) and divided using 2 × 13 g extrudate, 160 °C, 15 min heating only, and 10 min heating and pressing at 50 bar and cooling to 60 °C. This process is referred in the next text as a first thermal treatment.

Subsequently, a mini extruder (Wellzoom Desktop Extruder Line, Shenzhen, China) was used for the preparation of filaments from granulates using constant speed 10 cm·min^−1^, at two temperature zones 175 and 185 °C and air cooling. The average diameter of produced filaments was (1.75 ± 0.05) mm-typical filament dimensions processed by FFF technology. This process is referred in the next text as a second thermal treatment.

The used abbreviations of produced filaments are as follow: filament without additive–F_Ref, filament composed of PPR and synthetic aragonite–F_Ara_s, filament composed of PPR and synthetic calcite–F_Cal_s, filament composed of PPR and synthetic vaterite–F_Vat_s, filament composed of PPR and commercially available calcite–F_Cal_c. In the case of granulates, the abbreviations contain the letter G at the beginning instead of the letter F used for filaments.

### 2.3. Methods

Particle size distribution of used additives was recorded using laser granulometer LD 1090 (Cilas, Orléans, France). The measurements were performed in isopropyl alcohol, and each material was tested at least three times. BET specific surface area was measured using the device ASAP 2020 (Micromeritics, Norcross, GA, USA). The skeletal densities of the produced granulates and filaments were determined with a helium pycnometer AccuPyc II 1340 (Micromeritics, Lincoln, UK) using maximum pressure of 19.5 Psi and 10 cycles. The relative standard deviation of six replicates was calculated to be ≤0.05%. Viscoelastic properties of both granulates and filaments were characterized by a melt flow index (MFI) using extrusion plastometer M-201 (Chemoprojekt Praha, Czech Republic). The procedure, described in the ČSN EN ISO 1133 [53], was followed using these parameters of measurements: preheating load 240 s, test condition–*t* = 190 °C, load 2.16 kg, measuring length 10 mm, step length 0.25 mm, measurement starting time 300 s, die-diameter 2.095 mm, and length 8.00 mm. Produced filaments were characterized in terms of surface quality and surface roughness (*S*_a_–arithmetical mean roughness value and *S*_z_–mean roughness depth) using a Keyence VHX-6000 optical microscope (Keyence, Itasca, IL, USA) according to the standard ISO 25178 [54]. A caliper was used to measure filament diameters.

The tensile properties of the prepared filaments were determined using Instron 3345 (Instron, Norwood, MA, USA) with a maximum load of 5 kN and a constant load speed of 5 mm·min^−1^ following the standard CSN EN ISO 527-1 [55]. At least five replicates of each sample were tested.

Optical images of produced filaments were collected using an optical microscope Olympus TH4-200 (Olympus, Šindžuku, Japan). The morphology of used CaCO_3_ polymorphs and structural arrangements of produced filament was observed under a field emission scanning electron microscope (SEM) Quanta 450 FEG (FEI, Brno, Czech Republic) using a secondary electron detector. Observations were conducted at the 20 kV accelerating voltage. Powdered CaCO_3_ polymorphs were dispersed on carbon tape, as well as fragments of filament samples. Then, samples placed on stubs were coated with a 5-nm-thick layer of gold using a sputtering machine (Quorum Q150R ES, Quorum Technologies, Lewes, UK).

## 3. Results and Discussion

### 3.1. Characterization of Used CaCO_3_ Polymorphs

**Figure 1:**
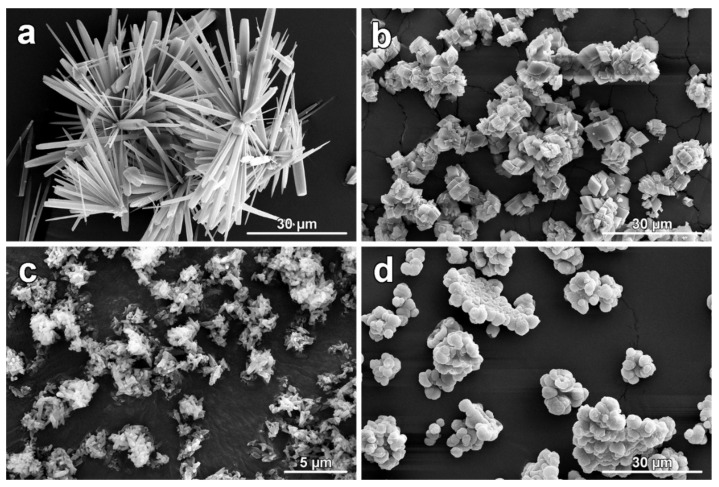
Collection of observed morphologies of synthesized CaCO_3_ polymorphs (aragonite (**a**), calcite (**b**), commercially available calcite (**c**), and vaterite (**d**)) observed under SEM.

In Figure 1, morphologies of used CaCO_3_ polymorphs observed under SEM are depicted. Synthetic aragonite formed needle-like crystals usually connected as larger clusters up to tens of µm (Figure 1a). Together with aragonite crystals, rhombohedral crystals of calcite were identified in smaller quantities. Synthetized calcite crystals were found to be present with typical euhedral to subhedral crystal habit. Larger calcite aggregates were composed mainly with the crystal of the size in the range from 1–3 µm and sporadically with crystal smaller than 0.5 µm (Figure 1b). Commercial calcite exhibited small and irregularly shaped crystals as a consequence of the grounding of raw limestone. The sizes of such particles varied from tens of nanometers to a few micrometers, usually with one elongated crystal site. As visible in Figure 1c, particles are tempted to be present in large aggregates up to several microns. Spherulitic crystals with a radius from 0.5–3.5 µm of synthetic vaterite built up from nanometric spherules (detail image reported in [56]) were detected (Figure 1d). In agreement with other CaCO_3_ particles, also vaterite crystals were observed to form larger (up to 10 µm) aggregates.

**Figure 2:**
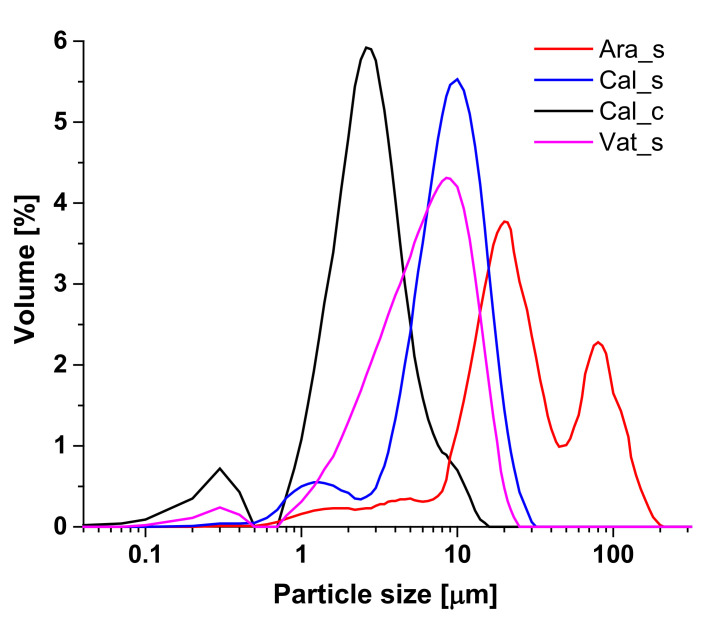
Comparison of recorded particle size distributions of CaCO_3_ polymorphs used as additives for filament production.

The particle size distribution detected in isopropanol suspensions is shown in Figure 2. As expected, the high difference in their shapes was recorded. Aragonite represents PSD with the largest particles of trimodal distribution with local maxima at 4, 20, and 85 µm. Bimodal distributions were found for the synthetic and commercial calcite with the local maxima at 1 and 10 µm and at 0.3 and 3 µm, respectively. In the case of synthetic vaterite, bimodal distribution with local maxima at 0.3 and 9 µm were recorded. It was illustrated by numerous investigations that morphology and particle size of CaCO_3_ have very high variability depending on the used reaction conditions and involvement of other chemicals [57]. Additionally, as mentioned in the Introduction, CaCO_3_ polymorphs show systems with tremendously different properties—e.g., hardness and reduced modulus. For example, reduced modulus of synthetized products (using the same reaction conditions as in this paper) was detected to be 5(4), 16(7), and 31(8) GPa calculated for aragonite, calcite, and vaterite, respectively [41]. The crystal morphologies of applied CaCO_3_ polymorphs, of course, also impacted the values of specific surfaces of their powders. The synthetic CaCO_3_ displayed values of 5.58 ± 0.02, 1.96 ± 0.01, and 2.34 ± 0.02 m^2^·g^−1^ for aragonite, calcite, and vaterite, respectively [41]. In the case of commercial calcite, the highest specific surface area of 6.73 ± 0.02 m^2^·g^−1^ was measured. The usage of additives with higher specific surface area values may result in their increased agglomeration within the polymer matrix. In the case of PP, the critical value of additives in polypropylene composite was identified to be 7 m^2^·g^−1^ [58], and it was shown that increased aggregations caused a significant decrease of strength and impact resistance.

### 3.2. Characterization of Prepared Granulates

**Figure 3:**
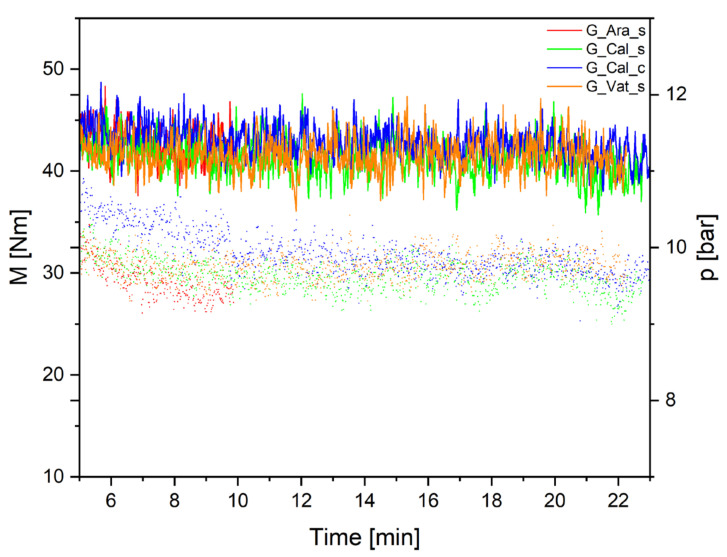
Dependence of torque *M* (full line) and head pressure *p* (dotted line) over time during granulate extrusion.

The progress of torque force (*M* [Nm]) and head pressure (*p* [bar]) during the extrudation of granulates is depicted in Figure 3. Constant values of torque over time (between 40–45 Nm) were recorded together with a slight decrease of the head pressure that oscillated between 7–10 bar after 10 min was reached. The G_Cal_c sample showed higher pressure values at the beginning of the processing in comparison with other samples. Such phenomena could be ascribed to the high specific surface area of applied commercial calcite particles, which may have had an increased tendency to partial agglomeration, nonetheless, after 11 min, pressure values started to be identical with the other samples. Thus, it can be assumed that suitable production conditions were achieved, and the Lumicene MR60MC2 PPR doped with the produced CaCO_3_ particles was successfully processed during extrusion.

The melt flow index (MFI) [59], reported in Table 1, represents a simple method for the characterization of rheological properties that play a crucial role in respect to the correct settings of the processing processes [60]. The lowest value was recorded for G_Ara_s, and others granulate showed comparable MFI values. Due to the fact that thermal degradation of CaCO_3_ occurs above ~600 °C [61], CaCO_3_ particles can retain the MFI value of PP and may improve the plasticity and processability of the polymers [62,63]. These properties are connected with the density of produced granulates. It can be seen in Table 1 that the addition of the CaCO_3_ particles resulted in the increase of density of around 4%, compared to the reference state.

### 3.3. Characterization of Prepared Filaments

**Figure 4:**
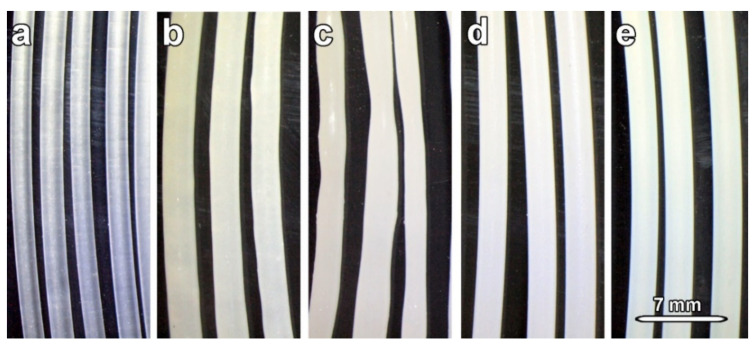
Photographic images of the prepared filaments: (**a**)—F_Ref, (**b**)—F_Ara_s, (**c**)—F_Cal_s, (**d**)—F_Cal_c, (**e**)—F_Vat_s.

The quality of the prepared filaments is very important for processing filaments using FFF technologies for 3D printing and has a major influence on trouble-free 3D printing, minimizing filament jams during winding, etc. Figure 4 shows the optical images of all prepared filaments. The F_Ref sample was produced as a transparent filament with a smooth surface without unevenness (Figure 4a). The additions of CaCO_3_ powders resulted in a whitish appearance (Figure 4b–e). The application of commercial calcite and synthetic vaterite resulted in products with comparable structures (Figure 4d,e). Only minor inequalities have been detected within the structure of filaments containing aragonite. The worst product quality was achieved in the case of the application of synthetic calcite. Such filaments showed an uneven thickness, which could have a negative effect during processing, and filament jams might be occurred during 3D printing in the extruder. Such behavior could be ascribed to physical incompatibility of Cal_s with some compounds presented in used PP random copolymer (Lumicene MR60MC as reported in Experimental Section). The diameter of REF sample was measured to be 1.60 ± 0.05 mm. The diameters of filaments containing additives were detected to be higher: 1.75 ± 0.05 mm for F_Ara_s, F_Cal_c, and F_Vat_s samples and 1.70 ± 0.10 mm in case of sample F_Cal_s.

**Figure 5:**
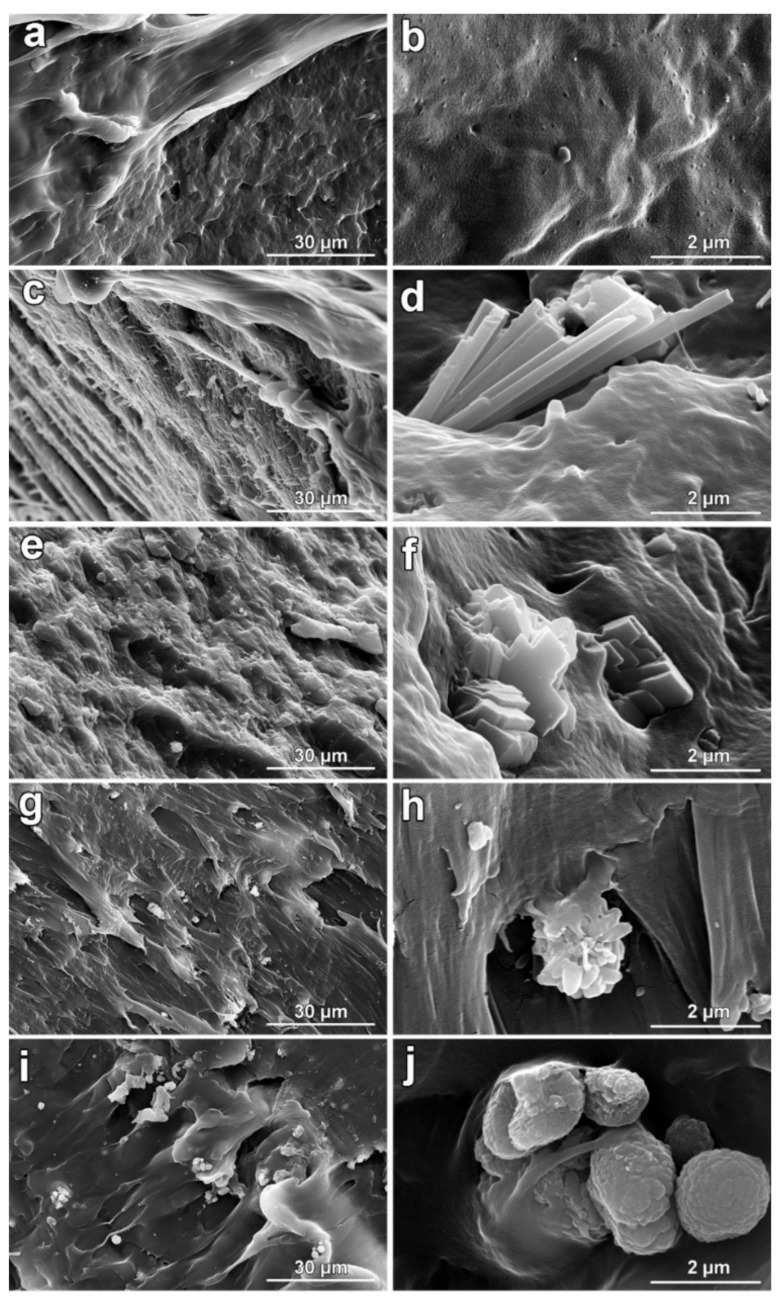
Collection of SEM images of prepared filaments collected at lower (**left**) and higher magnifications (**right**): (**a**,**b**)—F_Ref; (**c**,**d**)—F_Ara_s; (**e**,**f**)—F_Cal_s; (**g**,**h**)—F_Cal_c; (**i**,**j**)—F_Vat_s.

The collection of SEM images of the internal fragments of produced filaments of CaCO_3_ is shown in Figure 5. CaCO_3_ particles were found to be well dispersed within the PP matrix, especially in the case of filament contained with aragonite (Figure 5e), the needle-like particles are equally distributed in the same orientation, and it can be noted that during filament processing, the large clusters were disintegrated into much smaller objects. Unlike the others, filaments contained with synthetic calcite showed a rougher internal structure with the presence of larger structural disintegration areas up to a few hundred microns. Such observation may partially explain the worst quality of produced F_Cal_s filaments. Images collected at higher magnifications (Figure 5d,f,h,j), on the one hand, confirmed the disintegration of larger clusters of CaCO_3_ particles. On the other hand, the crystal’s habit of applied CaCO_3_ polymorphs (see Figure 1) during the filaments fabrication stayed preserved, even if it is visible (Figure 5d) that some of the aragonite’s needle-like crystals showed deformations. This behavior is the line with previous results [41] in which aragonite’s crystals were identified to be the most affected by thermal as well as pressure treatments. From the point of view of processing using FFF technology, the surface roughness of the prepared filaments is an important parameter. If high roughness values are reached, the filament becomes poorly processed, with a negative impact on winding. Thus, final products of inappropriate quality may be produced. As expected, different morphologies of applied CaCO_3_ polymorphs influenced the roughness values. The highest roughnesses, both *R*_a_ and *R*_z_, were detected in the case of filaments containing aragonite. Interestingly, filaments F_Cal_s, F_Cal_c, and F_Vat_s showed a lower roughness of *R*_a_ and *R*_z_ compared to the reference (Table 2). After the second thermal treatment during filament preparation, the MFI values—reported in Table 2—were found to be lower in comparison with the first thermal treatment used during the production of granulates (see Table 1), probably as a consequence of the further development of PP cross-linked structure [64]. Filaments containing CaCO_3_ particles with the highest specific surface area, commercial calcite, and synthetic aragonite showed almost similar MFI properties, and in comparison with the reference, MFI values were found to be lower for ca. 16%. It was observed [65] that the addition of lower content of CaCO_3_ with a smaller particle size tends to decrease the composite viscosity, which is related to the results of the MFI. The density of produced filaments containing CaCO_3_ particles was found to be slightly lower in comparison with values detected for granulates as a consequence of the development of structural cross-linking [64].

The results of the mechanical performance of produced filaments are summarized in Table 3. Obtained values of tensile stresses differs according to the presence of specific CaCO_3_ particles. It was shown in [66] that different morphologies of CaCO_3_ affected tensile strengths of tested powders. It can be noted that filaments produced with CaCO_3_ particles showed, in most cases, a reduction of mechanical properties. The only exception is filament fabricated with aragonite. In this case, the mechanical properties were found to be improved—e.g., tensile stress was approx. 12% higher. The explanation may be found in the crystal habit of aragonite particles. Aragonite’s needle-like particles that were observed to be homogenously and unidirectionally orientated within the PP matrix (Figure 5c,d) may act as reinforced material that positively affected mechanical performance. Reinforced materials are commonly used to enhance mechanical properties of, e.g., concretes [67,68]. From the rest of the three applied types of CaCO_3_ particles, the filaments F_Cal_c and F_Vat_s showed comparable properties, and with respect to F_Ref, the only small decrease was recorded. However, in the case of F_Cal_s, a huge drop in mechanical performance was recorded (more than 50% in the comparison of tensile stress and Young modulus of F_Ref). It is not unexpected due to the bad quality of produced filament F_Cal_c (see Figure 4c) with tapering segments that have a strong impact on tensile stress. Comparison with the literature is difficult due to the lack of data focus on the products needed for 3D printing. In different systems, CaCO_3_ particles have been used for the PP surface modifications [69,70], and the role of CaCO_3_ on the crystallization of PP was investigated [71]. The usage of nanocalcite with a particle size of 70 nm showed an improving effect on Young’s modulus, tensile yield stress, and impact strength [72,73]. Aragonite was recognized to have a more beneficial effect than calcite as filler in polyvinyl chloride or polypropylene [74].

## 4. Conclusions

The presented study has shown the possibilities of the application of CaCO_3_ particles for the fabrication of polypropylene filaments employed for 3D printing. Moreover, the effect of the usage of different anhydrous crystalline CaCO_3_ polymorphs on the physical-mechanical properties of produced granulates and filaments containing additives has been investigated employing the combination techniques. Microscopic observations showed tremendously different crystal habits of applied CaCO_3_ particles that resulted in specific particle size distributions and specific surface areas. PP granulates with additions of CaCO_3_ have been processed without significant differences and produced granulates showed approximately 4% higher densities compared to the reference sample. Next, heat treatment, production of filaments caused a decrease of MFI and density values as a consequence of more connected cross-linked structures. In the case of filaments produced with the addition of synthetic calcite, the resulting filaments showed crooked structure in contrast with other samples. Microscopic observations showed a homogenous distribution of CaCO_3_ particles, especially in the case of aragonite crystals. Different physical properties of produced filaments have been reflected in their mechanical performance.

In comparison with the reference sample, a decrease of tensile stress values has been measured, with one exception—filaments with synthetic aragonite. In this case, tensile stress was found to be higher for 12%. This behavior is explained by homogenous and unidirectional dispersion of aragonite’s particles within the PP matrix and the ability of needle-like aragonite crystals to act as reinforced material, commonly used in the cement industry to improve mechanical performance. The produced CaCO_3_ filaments may find their applications in 3D printing, and our next study will be focused on the characterization of printed products employing produced novel CaCO_3_ filaments.

## Figures and Tables

**Table 1:** Overview of determined physical properties of produced granulates (calculated standard deviations are reported in the brackets).

Sample	MFI (g·10 min^−1^)	Density (g·cm^−3^)
G_Ref	27.0(1)	0.8962(3)
G_Ara_s	24.0(2)	0.9330(4)
G_Cal_s	25.7(6)	0.9362(6)
G_Cal_c	26.0(6)	0.9368(4)
G_Vat_s	26.1(2)	0.9312(5)

**Table 2:** Overview of determined physical properties of produced granulates (calculated standard deviations are reported in the brackets).

Sample	MFI (g·10 min^−1^)	*R*_a_ (µm)	*R*_z_ (µm)	Density (g·cm^−3^)
F_Ref	22.7(2)	7.8(5)	42(1)	0.8979(8)
F_Ara_s	19.0(1)	11.0(1)	55(1)	0.9291(7)
F_Cal_s	23.9(1)	5.7(5)	28(1)	0.9290(1)
F_Cal_c	20.0(1)	4.8(4)	28(1)	0.9320(6)
F_Vat_s	25.1(4)	5.7(8)	38(1)	0.9285(4)

**Table 3:** Summarization of determined mechanical properties of prepared filaments.

Sample	Max. Load (N)	Tensile Stress (MPa)	Young Modulus (MPa)	Tensile Deformation (%)
F_Ref	34.6 ± 4.4	24.5 ± 1.7	1187.1 ± 73.2	4.5 ± 0.7
F_Ara_s	38.3 ± 3.7	27.4 ± 2.0	1074.1 ± 70.8	4.8 ± 0.5
F_Cal_s	19.0 ± 2.7	11.3 ± 3.5	459.6 ± 73.3	3.5 ± 0.7
F_Cal_c	32.5 ± 3.9	20.9 ± 1.8	765.2 ± 67.0	5.4 ± 0.4
F_Vat_s	29.8 ± 3.4	21.5 ± 1.1	811.8 ± 118.8	5.1 ± 0.8

## Data Availability

Not applicable.
